# MELK as a Mediator of Stemness and Metastasis in Aggressive Subtypes of Breast Cancer

**DOI:** 10.3390/ijms26052245

**Published:** 2025-03-03

**Authors:** Breanna McBean, Reine Abou Zeidane, Samuel Lichtman-Mikol, Benjamin Hauk, Johnathan Speers, Savannah Tidmore, Citlally Lopez Flores, Priyanka S. Rana, Courtney Pisano, Meilan Liu, Alyssa Santola, Alberto Montero, Alan P. Boyle, Corey W. Speers

**Affiliations:** 1 Department of Human Genetics, University of Michigan, Ann Arbor, MI 48109, USA; bmcbean@umich.edu (B.M.); apboyle@umich.edu (A.P.B.); 2 Department of Computational Medicine and Bioinformatics, University of Michigan, Ann Arbor, MI 48109, USA; 3 Department of Radiation Oncology, University Hospitals Case Medical Center, Cleveland, OH 44106, USA; rxa619@case.edu (R.A.Z.); sxl2230@case.edu (S.L.-M.); bxh462@case.edu (B.H.); jwspeers103@gmail.com (J.S.); stidmore@laurelschool.org (S.T.); lopezc43@unlv.nevada.edu (C.L.F.); pxr240@case.edu (P.S.R.); courtney.pisano@uhhospitals.org (C.P.); alberto.montero@uhhospitals.org (A.M.); 4 Department of Radiation Oncology, Case Western Reserve University, Cleveland, OH 44106, USA; 5 Department of Radiation Oncology, University of Michigan, Ann Arbor, MI 48109, USA; lmiu@med.umich.edu (M.L.); ajsantola@gmail.com (A.S.)

**Keywords:** MELK, triple-negative breast cancer (TNBC), metastasis, invasion, migration, tumor initiation

## Abstract

Triple-negative breast cancer (TNBC) is the breast cancer subtype with the poorest prognosis and lacks actionable molecular targets for treatment. Maternal embryonic leucine zipper kinase (MELK) is highly expressed in TNBC and has been implicated in poor clinical outcomes, though its mechanistic role in the aggressive biology of TNBC is poorly understood. Here, we demonstrate a role of MELK in TNBC progression and metastasis. Analysis of publicly available datasets revealed that high *MELK* expression correlates with worse overall survival, recurrence-free survival, and distant metastasis-free survival, and *MELK* is co-expressed with metastasis-related genes. Functional studies demonstrated that MELK inhibition, using genomic or pharmacologic inhibition, reduces mammosphere formation, migration, and invasion in high-MELK-expressing TNBC cell lines. Conversely, MELK overexpression in low-MELK-expressing cell lines significantly increased invasive capacity in vitro and metastatic potential in vivo, as evidenced by enhanced metastasis to the liver and lungs in a chorioallantoic membrane assay. These findings highlight MELK as a key regulator of TNBC aggressiveness and support its potential as a therapeutic target to mitigate metastasis and improve patient outcomes.

## 1. Introduction

Breast cancer remains the most commonly diagnosed invasive cancer among women in the United States and is the second leading cause of cancer-related mortality [1]. Advances in early detection and treatment have improved outcomes for many subtypes of breast cancer; however, disparities persist, particularly for aggressive subtypes. Breast cancer is not a single disease, but a heterogeneous group of malignancies defined by the presence or absence of molecular markers such as estrogen receptor (ER), progesterone receptor (PR), and HER2 amplification. These markers guide the development of targeted therapies, which have significantly improved survival in patients with hormone receptor-positive or HER2-positive breast cancers. Unfortunately, 10–25% of breast cancer cases lack these markers, classifying them as triple-negative breast cancer (TNBC), a subtype associated with poor prognosis and limited treatment options [2,3].

TNBC is characterized by its rapid progression, early metastasis, and marked heterogeneity both within and between tumors [4,5,6,7]. Compared to other breast cancer subtypes, TNBC exhibits a higher proportion of breast cancer stem cells (BCSCs), which are associated with tumor initiation, therapeutic resistance, and metastasis [8]. These factors, coupled with the absence of actionable molecular targets, contribute to TNBC’s aggressive nature and make it one of the most challenging breast cancer subtypes to treat effectively. Current treatment strategies including chemotherapy, surgery, and radiotherapy are often initially effective, but rates of disease recurrence and metastasis are higher in women with TNBC, underscoring the urgent need for novel therapeutic targets [4,5].

Maternal embryonic leucine zipper kinase (MELK) has emerged as a potential driver of aggressive cancer phenotypes and a promising therapeutic target in TNBC. MELK is a serine/threonine kinase that is overexpressed in multiple malignancies, including breast cancer, where its expression is particularly high in TNBC compared to ER-positive subtypes [9,10,11]. High MELK expression correlates with poor overall survival, shorter recurrence-free survival, and increased likelihood of distant metastasis [10,11,12]. Functionally, MELK has been implicated in regulating cell cycle progression, stem cell maintenance, and metastatic behavior [10]. These roles are supported by MELK’s interactions with oncogenic pathways, such as FOXM1 activation and NF-κB signaling, which are critical in TNBC pathobiology [10,13,14,15]. Furthermore, its minimal expression in normal tissues makes MELK an attractive target for cancer-specific interventions [11,16].

Given the aggressive nature of TNBC and the lack of effective targeted therapies, there is significant interest in exploring MELK as a potential therapeutic target. Building on previous studies that highlight MELK’s role in tumorigenesis, radiation resistance, and metastasis, we sought to further characterize its functional relevance in TNBC [10,17]. In this study, we integrate clinical data analyses with preclinical models to examine the impact of *MELK* expression on survival, tumor-initiating properties, and metastatic potential. Specifically, we assess how MELK knockdown and pharmacological inhibition affect cancer stem cell properties, migration, and invasion; and how MELK overexpression influences metastatic capacity in vivo. These findings aim to provide a foundation for the development of MELK-targeted therapies that could improve outcomes for patients with this aggressive breast cancer subtype.

## 2. Results

### 2.1. High MELK Expression Correlates with Poor Survival Outcomes in Breast Cancer

**Figure 1:**
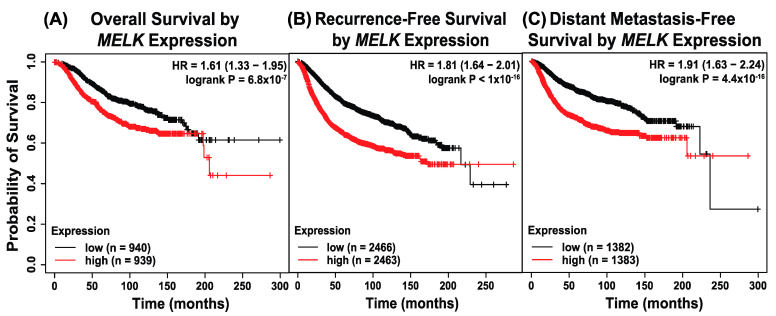
High *MELK* expression is associated with significantly worse survival. Using a large publicly available data source (KMplot.com), the effect of *MELK* expression on various survival metrics was measured. *MELK* expression was stratified by median expression, with higher and lower than median expression evaluated. (**A**) Overall survival, (**B**) recurrence-free survival, and (**C**) distant metastasis-free survival were compared with HR and *p*-values depicted. HR: Hazard ratio.

Kaplan–Meier survival analyses were conducted on data from the Kaplan–Meier Plotter database for breast cancer stratified by median *MELK* expression to evaluate the impact of *MELK* expression on clinical outcomes, including overall survival (OS), recurrence-free survival (RFS), and distant metastasis-free survival (DMFS), in breast cancer patients [18]. Breast cancer gene expression and clinical outcome data were assembled for 7830 unique tumor samples from 55 independent datasets. Relapse-free survival was available for 5268 patients and overall survival time for 5165 patients [18]. Clinical characteristics for the entire database were described previously, including receptor status, grade, lymph node status, molecular subtype distribution, applied treatment, and length of follow-up for relapse-free survival [18]. Patients with higher than median *MELK* expression exhibited significantly worse survival across all metrics compared to those with lower than median *MELK* expression. For OS (n = 1879), the median survival was 66.12 months (5.5 years) in the high-expression cohort versus 136.80 months (11.4 years) in the low-expression cohort (Figure 1A). Similarly, for RFS (n = 4929), the median time to recurrence was 171.43 months (14.3 years) for the high-expression cohort compared to 216.66 months (18.1 years) for the low-expression cohort (Figure 1B). For DMFS (n = 2765), the high-expression cohort had a median distant metastasis-free time of 41.64 months (3.5 years), compared to 137.00 months (11.4 years) in the low-expression cohort (Figure 1C). These data indicate that elevated *MELK* expression is strongly associated with poorer survival outcomes in breast cancer.

**Figure 2:**
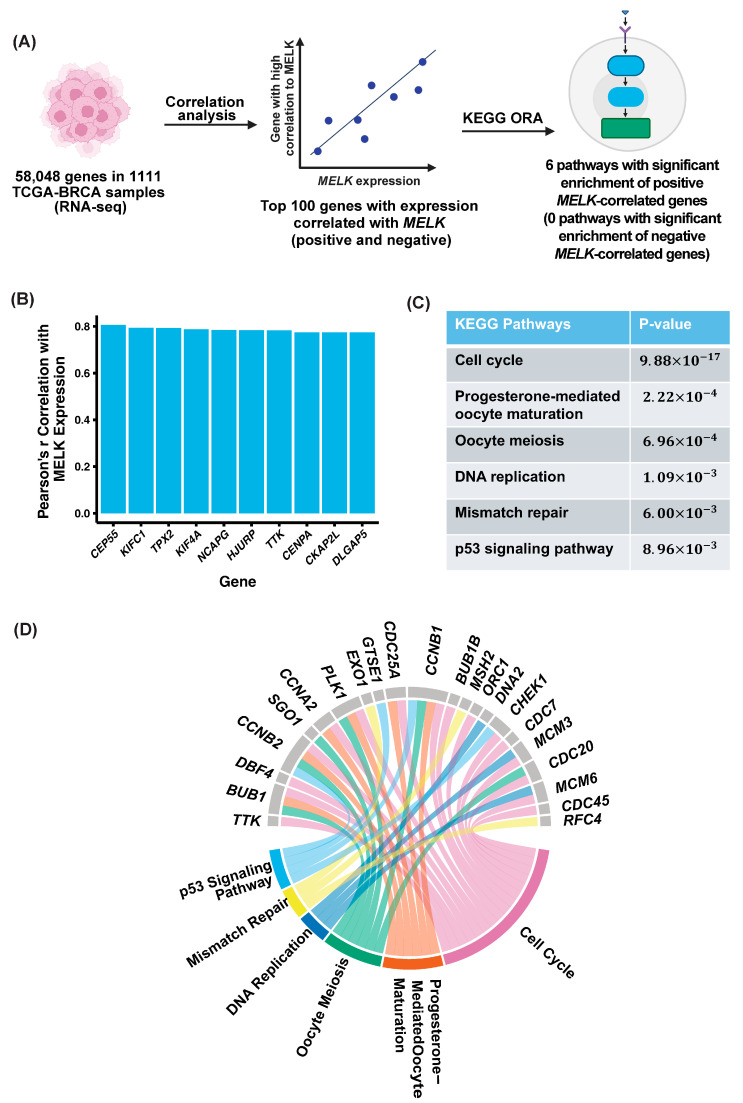
MELK and correlated gene expression are associated with tumorigenesis and metastasis pathways. (**A**) Schema of pathway analysis. Using gene expression from the TCGA-BRCA breast dataset, the top 100 genes positively and negatively correlated with *MELK* expression were used for overrepresentation analysis (ORA) in pathways from the Kyoto Encyclopedia of Genes and Genomes (KEGG). (**B**) A bar chart depicting the top ten genes with expression most positively correlated with *MELK* expression. (**C**) This resulted in six pathways from the positively correlated genes, shown in this table. *p*-values reported are adjusted. (**D**) A chord diagram of the pathways (colored borders) and the significantly correlated genes (grey borders) involved in each of the pathways. Genes are ordered by decreasing Pearson’s r in the clockwise direction and pathways by decreasing pathway significance in the clockwise direction. There were no significant pathways among the negatively correlated genes.

To explore potential mechanisms underlying the association between *MELK* expression and poor prognosis, we analyzed the transcriptomic profiles of primary breast cancer tumors from the TCGA-BRCA database (n = 1111). This analysis, as described in the methods, identified 14,503 genes significantly positively correlated and 3544 genes significantly negatively correlated with *MELK* expression (Figure 2A; top 100 genes, Pearson’s r, and *p*-values for positive and negative correlations in Supplemental Tables S1 and S2, respectively). Notably, among the top ten genes most positively correlated with *MELK* expression were *CEP55* (Pearson’s r = 0.806, *p* < 1 × 10^−16^), *KIFC1* (Pearsons’s r = 0.794, *p* < 1 × 10^−16^), *TPX2* (Pearson’s r = 0.793, *p* < 1 × 10^−16^), *KIF4A* (Pearson’s r = 0.778, *p* < 1 × 10^−16^), *NCAPG* (Pearson’s r = 0.785, *p* < 1 × 10^−16^), *HJURP* (Pearson’s r = 0.783, *p* < 1 × 10^−16^), *TTK* (Pearson’s r = 0.783, *p* < 1 × 10^−16^), *CENPA* (Pearson’s r = 0.774, *p* < 1 × 10^−16^), *CKAP2L* (Pearson’s r = 0.774, *p* < 1 × 10^−16^), and *DLGAP5* (Pearson’s r = 0.774, *p* < 1 × 10^−16^), which have all been previously associated with tumor progression and metastasis (Figure 2B). *CEP55* expression is correlated with metastasis in breast cancer [19] and esophageal cancer [20]; *KIFC1* is found in non-small-cell lung cancer [21] and head and neck cancers [22]; *TPX2* in prostate cancer [23] and non-small-cell lung cancer [24]; *KIF4A* in colorectal cancer [25] and liver cancer [26]; *NCAPG* in non-small-cell lung cancer [27]; *HJURP* in liver cancer [28] and pancreatic cancer [29]; *TTK* in endometrial cancer [30] and breast cancer [31]; *CENPA* in clear renal cell carcinoma [32]; *CKAP2L* in clear renal cell carcinoma [33]; and *DLGAP5* in bladder cancer [34]. These findings suggest that MELK may contribute to poor clinical outcomes in breast cancer through its association with genes involved in oncogenic pathways and may be associated with increased metastatic potential.

To determine the biological processes associated with genes correlated with *MELK* expression, we conducted overrepresentation analysis (ORA) of the top 100 positively and 100 negatively correlated genes (Supplemental Tables S1 and S2). Kyoto Encyclopedia of Genes and Genomes (KEGG) pathway analysis revealed no significant enrichment among negatively correlated genes. However, six pathways were significantly enriched among positively correlated genes: cell cycle (*p* = 9.88 × 10^−17^), progesterone-mediated oocyte maturation (*p* = 2.22 × 10^−4^), oocyte meiosis (*p* = 6.96 × 10^−4^), DNA replication (*p* = 1.09 × 10^−3^), mismatch repair (*p* = 6.00 × 10^−3^), and p53 signaling pathway (*p* = 8.96 × 10^−3^) (Figure 2C,D). These pathways align with MELK’s established role in cell cycle regulation, particularly at the G2/M transition, where it phosphorylates key regulatory proteins. Furthermore, MELK’s activation of FOXM1, a transcription factor that drives the expression of mitotic regulatory proteins, is consistent with these findings [13]. These data suggest that MELK-associated genes contribute to critical cellular processes involved in tumorigenesis, including cell division, DNA replication, and genomic stability.

Furthermore, correlated genes in three or more of these pathways include *BUB1*, *CCNB2*, *PLK1*, and *CCNB1*. *BUB1* has been implicated as a stem cell regulator in breast cancer [35]. Additionally, *CCNB2* has been linked to metastasis in nasopharyngeal cancer [36], breast cancer [37], and non-small-cell lung cancer [38]; *PLK1* in pancreatic cancer [39] and melanoma [40]; and *CCNB1* in esophageal cancer [41]. So, here, we have shown that genes co-expressed with *MELK* in breast cancer tumors have previously been implicated in metastasis. Additionally, MELK has previously been associated with stem cell maintenance in neural stem cells in mouse development and glioblastoma stem cells [42] and metastasis in lung cancer [43], esophageal cancer [15], oral cancer [44], and even triple-negative breast cancer [10]. Thus, we next decided to further investigate the impact of MELK on stem cell properties and metastasis in breast cancer.

### 2.2. Knockdown or Inhibition of MELK Reduces Mammosphere Formation Efficiency and Growth

**Figure 3:**
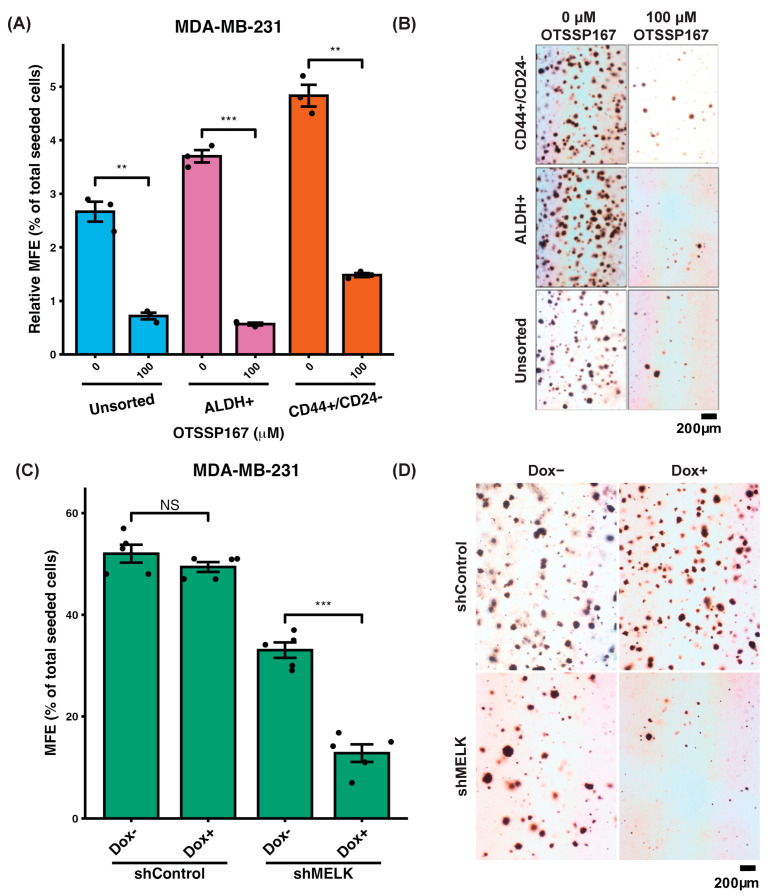
MELK inhibition reduces mammosphere formation efficiency in high-MELK-expressing MDA-MB-231 cells. (**A**) Treatment with the MELK inhibitor OTSSP167 significantly reduced mammosphere formation efficiency in unsorted cells (n = 3, *p* = 0.0049), ALDH+ cancer stem cells (n = 3, *p* = 0.0008), and CD44+/CD24− cancer stem cells (n = 3, *p* = 0.00282). Each condition was normalized to unsorted cells. (**B**) Representative images of mammospheres for each sorted group under control and the treatment conditions at 10× magnification. (**C**) Doxycycline (Dox)-inducible shRNA against MELK (shMELK) in CD44+/CD24− MDA-MB-231 cells significantly reduced mammosphere formation efficiency (MFE) after induction (n = 5, *p* = 2.4 × 10^−5^), with no significant change observed in cells expressing control shRNA (shControl, n = 5, *p* = 0.239). (**D**) Representative photographs of mammospheres formed in shMELK and shControl conditions at 10× magnification. Data represent the mean of independent experiments, with error bars indicating SEM. Statistical comparisons were performed using a 2-sided Student’s *t*-test. ** *p* < 0.01, *** *p* < 0.001, NS *p* ≥ 0.05. Images were taken at 10× magnification.

There is support for MELK’s role in the maintenance of stem and progenitor cells during development in mice [42] and zebrafish [45]. We used a mammosphere formation assay to assess the role of MELK in stem cell properties in breast cancer. We evaluated the role of MELK in breast cancer stem cell properties using a mammosphere formation assay in MDA-MB-231 cells, a high-MELK-expressing cell line. Treatment with the MELK inhibitor OTSSP167 significantly reduced mammosphere formation efficiency in cancer stem cell populations (CD44+/CD24− and ALDH+) compared to untreated controls. A less pronounced decrease in mammosphere formation was observed in the unsorted population, which largely contains non-stem cells, upon OTSSP167 treatment, further supporting MELK’s involvement in maintaining cancer stem cell properties (Figure 3A, representative images in Figure 3B) [46].

To corroborate the pharmacological findings, we performed shRNA-mediated knockdown of MELK in CD44+/CD24− MDA-MB-231 cells. Stable cell lines expressing doxycycline (dox)-inducible MELK shRNA (shMELK) or a dox-inducible non-targeting control shRNA (shControl) were generated. Effective MELK knockdown in shMELK cells was validated by western blot and qPCR, while no knockdown was observed in shControl cells (Supplemental Figure S1A,B). Upon doxycycline induction, mammosphere formation efficiency was reduced by approximately 50% in shMELK cells, with no significant change in shControl cells (Figure 3C; representative images in Figure 3D). These results indicate that MELK plays a critical role in tumor initiation and clonogenicity, a key stem cell property observed in breast cancer.

### 2.3. Knockdown of MELK Reduces Invasion In Vitro and Metastasis In Vivo

**Figure 4:**
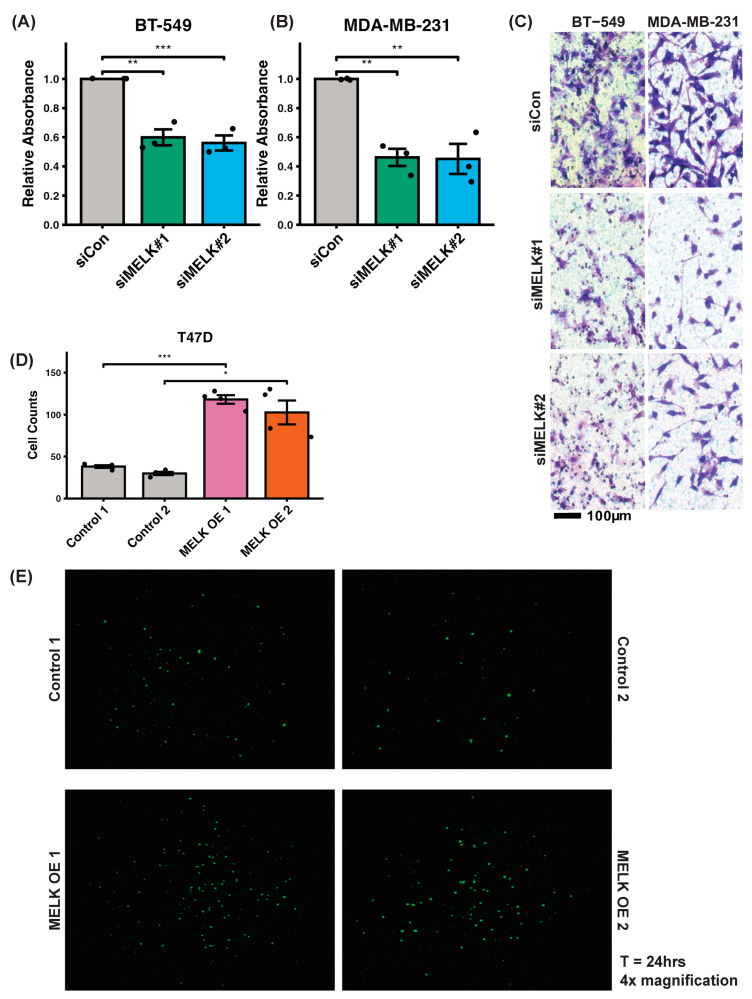
MELK knockdown reduces invasion efficiency in high-MELK-expressing cell lines BT-549 and MDA-MB-231, while MELK overexpression increases invasion efficiency in low-MELK-expressing T47D cells. (**A**) siRNA-mediated MELK knockdown significantly reduced invasion in BT-549 cells (n = 3; *p* = 0.0011 for siMELK#1, *p* = 0.0007 for siMELK#2). (**B**) Similar reductions in invasion were observed in MDA-MB-231 cells (n = 3; *p* = 0.0026 for siMELK#1, *p* = 0.0023 for siMELK#2). (**C**) Representative images of cells which successfully invaded the membrane for both cell lines. Images were taken at 40× magnification. (**D**) MELK overexpression in T47D cells (MELK OE 1 and MELK OE 2) significantly increased invasion efficiency compared to LacZ controls (Control 1 and Control 2; n = 4; *p* = 0.0002 and *p* = 0.0135 for Control 1/MELK OE 1 and Control 2/MELK OE 2, respectively). (**E**) Representative images of invaded cells for T47D control and MELK-overexpressing clones. Images were taken at 4× magnification. Data represent the mean of independent experiments with error bars indicating SEM. Statistical comparisons were performed using (**A**,**B**) one-way ANOVA with Dunnetts Multiple Comparison Test and (**D**) 2-sided Student’s *t*-test. * *p* < 0.05, ** *p* < 0.01, *** *p* < 0.001. OE: Overexpression.

Given MELK’s reported association with metastasis in various cancers [10,15,43,44], we investigated its role in invasion using the Fluoroblok Tumor Invasion Assay, modified to measure absorbance from stained cells. Two siRNAs targeting MELK (siMELK#1 and siMELK#2) were employed to knock down MELK expression in two high-MELK-expressing TNBC cell lines, BT-549 and MDA-MB-231. Successful MELK knockdown was confirmed via western blot (Supplemental Figure S2A). In BT-549 cells, both siMELK#1 and siMELK#2 reduced invasion efficiency by approximately 40% compared to the control siRNA (siCon) (Figure 4A). Similarly, in MDA-MB-231 cells, MELK knockdown resulted in over a 50% reduction in invasion efficiency (Figure 4B). Representative images of cells which invaded the membrane in these experiments also illustrate the diminished invasion capacity in MELK-knockdown cells compared to control, as we see fewer stained cells in the siMELK#1 and siMELK#2 treated cells than in the siCon control images (Figure 4C). These findings demonstrate that MELK is a critical regulator of invasion in high-MELK-expressing breast cancer cell lines.

### 2.4. Overexpression of MELK Increases Invasion Efficiency In Vitro

To determine whether overexpressing MELK in a cell line with low endogenous MELK expression could enhance invasive capacity, we generated T47D cells that stably overexpress MELK through lentiviral transduction of MELK-GFP. Successful MELK overexpression was confirmed via western blot analysis (Supplemental Figure S2B). Compared to LacZ control cells (Control 1 and Control 2), two independent MELK-overexpressing clones (MELK OE 1 and MELK OE 2) exhibited a more than twofold increase in invasive capacity, as evidenced by the number of cells that traversed the membrane (fewer than 50 cells for both controls versus over 100 cells for both overexpression clones; Figure 4D). Representative images of cells which invaded the membrane also show this enhanced invasion capacity with MELK overexpression in a low-MELK-expressing cell line, as evidenced by the increased number of fluorescent cells in the MELK OE 1 and MELK OE 2 conditions relative to the Control 1 and Control 2 conditions (Figure 4E). These findings, in conjunction with the knockdown experiments, provide evidence of MELK’s role in invasiveness in breast cancer.

### 2.5. Inhibition of MELK Reduces Migration In Vitro

**Figure 5:**
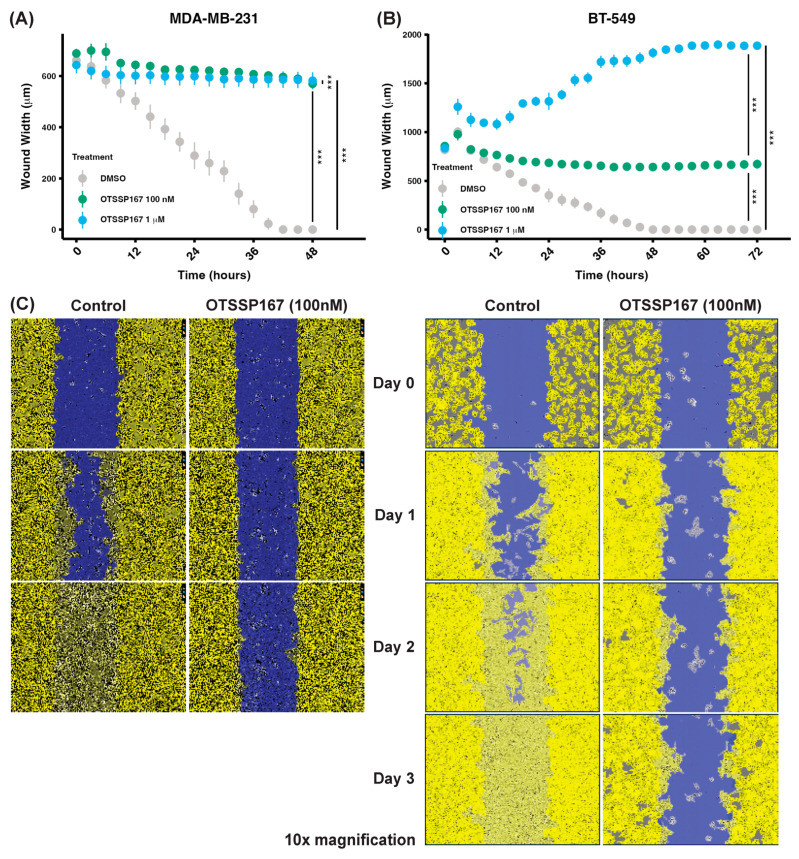
MELK inhibition reduces migration in high-MELK-expressing cell lines MDA-MB-231 and BT-549. (**A**) Wound width over time in MDA-MB-231 cells treated with DMSO (control) or the MELK inhibitor OTSSP-167 at 100 nM and 1 μM (n = 12). (**B**) Wound width over time in BT-549 cells under the same treatment conditions (n = 12). (**C**) Representative images of wound closure at selected time points, including masks generated by the Incucyte system for quantification at 10× magnification (MDA-MB-231, left; BT-549 right). Data demonstrate the dose-dependent effect of OTSSP-167 on migration in high-MELK-expressing cells. Error bars represent standard deviation. Statistical comparisons were performed using a one-way repeated-measures ANOVA with post-hoc pairwise *t*-tests to assess differences between each treatment group. *** *p* < 0.001.

To evaluate the impact of MELK inhibition on cellular migration, we performed scratch-wound assays in two high-MELK-expressing cell lines, MDA-MB-231 and BT-549, treated with the MELK inhibitor OTSSP167 at concentrations of 100 nM and 1 μM. In MDA-MB-231 cells, treatment with OTSSP167 significantly impaired wound closure at both concentrations compared to the control (*p* < 0.0001; Figure 5A, representative images in Figure 5C). A dose-dependent reduction in migration was also evident, with significant differences between 100 nM and 1 μM treatments (*p* = 0.0005). Similarly, in BT-549 cells, both concentrations of OTSSP167 significantly reduced wound closure compared to the DMSO control (1 μL/mL) (*p* < 0.0001 for both concentrations; Figure 5B, representative images in Figure 5C). Furthermore, a dose-dependent effect was observed, with significant differences in wound closure between 100 nM and 1 μM OTSSP167 treatments (*p* < 0.0001). Collectively, these results demonstrate that MELK inhibition impairs migration efficiency in vitro in high-MELK-expressing breast cancer cell lines.

### 2.6. Overexpression of MELK Increases Metastasis In Vivo

**Figure 6:**
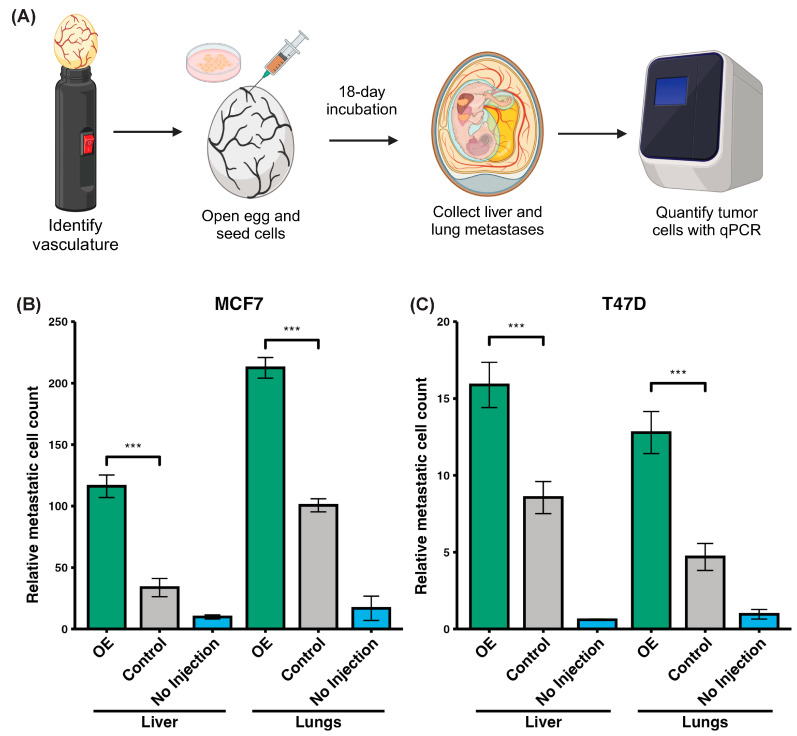
*MELK* overexpression in low-*MELK*-expression cell lines, MCF7 and T47D, increases metastasis in the chorioallantoic membrane (CAM) assay. (**A**) Schematic of the CAM assay and experimental design. (**B**) *MELK* overexpression (OE) in MCF7 cells resulted in a significant increase in metastatic cells in the liver (n = 10, *p* < 0.0001) and the lung (n = 10, *p* < 0.0001). (**C**) Similarly, *MELK* overexpression in T47D cells resulted in a significant increase in metastatic cells in the liver (n = 10, *p* < 0.0001) and the lung (n = 10, *p* < 0.0001). Data represent 10 independent experiments, and error bars represent SEM. A 2-sided Student’s *t*-test was used for comparison. *** *p* < 0.001.

To assess whether *MEL*K overexpression promotes metastasis in vivo, we utilized the chorioallantoic membrane (CAM) assay (Figure 6A). Stable *MELK*-overexpressing cell lines were generated using two low-MELK-expressing cell lines, MCF7 and T47D (*MELK* overexpression confirmed by qRT-PCR in Supplemental Figure S3). In MCF7 cells, *MEL*K overexpression significantly increased metastatic cell counts in the liver, with a mean of 116.07 metastatic cells compared to 33.70 in the control—a 3.5-fold increase. Similarly, in the lungs, the mean number of metastatic cells rose from 100.63 in the control group to 212.55 in the *MELK*-overexpressing group, more than doubling the metastasis rate and number (Figure 6B). Comparable results were observed in T47D cells, where *MEL*K overexpression nearly doubled the mean metastatic cell count in the liver (8.55 to 15.89) and nearly tripled the count in the lungs (4.69 to 12.79; Figure 6C). Collectively, these results from two distinct cell line models strongly indicate that *MELK* overexpression enhances metastatic potential in breast cancer.

## 3. Discussion

Our study demonstrates that maternal embryonic leucine zipper kinase (MELK) plays a critical role in the aggressive behavior of triple-negative breast cancer (TNBC). High *MELK* expression correlates with worse overall survival, recurrence-free survival, and distant metastasis-free survival (Figure 1), aligning with previous studies that have identified MELK as a negative prognostic factor in TNBC and other cancers. *MELK* is also co-expressed with other genes which have been implicated in metastasis in a publicly available dataset (Figure 2). Using both genetic (shRNA) and pharmacologic (OTSSP167) knockdown of MELK in high-MELK-expressing cell lines, we showed that MELK inhibition reduces mammosphere formation efficiency (Figure 3). Reducing MELK expression in these cell lines also reduced invasion (Figure 4) and migration efficiency (Figure 5) in vitro. We also showed that overexpression of MELK in a low-MELK-expressing cell line increased invasion efficiency in vitro (Figure 4), and in an in vivo system, *MELK* overexpression resulted in increased metastases in the liver and lungs (Figure 6). Altogether, these data demonstrate that MELK inhibition is a potentially effective strategy for reducing metastasis in TNBC, which may ultimately lead to better patient prognoses.

Maternal embryonic leucine zipper kinase (MELK) has emerged as a potential driver of progression and metastasis of various cancers, including triple-negative breast cancer (TNBC) [47]. MELK is a serine/threonine kinase that is overexpressed in several cancer types and is associated with poor prognosis and aggressive tumor behavior [9,10,11,12,13,14,15,43,44,48,49,50,51,52]. There is evidence in the literature that this overexpression in TNBC tumors is caused by either copy number gains in this subtype [53] or mutant p53 activity [54]. The reexpression of MELK in TNBC, as opposed to normal tissues, is hypothesized to be driven by these genetic changes that reactivate embryonic pathways involved in cell cycle regulation and survival, crucial for cancer cell proliferation and resistance to apoptosis. Furthermore, mutations in p53 have been shown to induce increased expression of MELK by release of wild type p53 suppression of FOXM1 [54]. As the majority of TNBC tumors harbor mutations in p53 (compared to ER+ BC and normal tissues), this may explain the increased expression noted in TNBC [55]. In cancer, MELK is involved in regulating cell cycle progression, apoptosis, and proliferation. Specifically, MELK has been shown to promote tumorigenesis by phosphorylating and activating key proteins involved in cell division and survival, such as FOXM1, which drives the expression of genes critical for mitosis [13,14,56]. MELK has also been shown to influence radiation and chemotherapy response and is an important determiner of intrinsic breast cancer subtype [17,57,58,59]. Previous studies have shown that MELK expression correlates with increased metastatic potential and poor outcomes in cancers such as lung, esophageal, and oral squamous cell carcinoma [15,43,44]. The kinase facilitates metastasis by enhancing cell migration and invasion, at least in part, through the modulation of pathways such as NF-κB, which is known to promote metastasis and epithelial–mesenchymal transition (EMT) [10,15].

While our study offers significant insights into MELK’s role in TNBC, there are important limitations to consider. First, while our study utilizes both genetic and pharmacological approaches to inhibit MELK, the potential off-target effects of these methods cannot be entirely ruled out. Specifically, the use of shRNA and pharmacological inhibitors such as OTSSP167, which may have additional targets beyond MELK, could confound the interpretation of the results. Second, although the CAM assay provides a physiologically relevant in vivo model, it does not fully recapitulate the tumor microenvironment of human breast cancer, particularly immune and stromal interactions. Expanding these findings into patient-derived xenograft (PDX) models and incorporating immune-competent systems will enhance translational relevance. Additionally, TNBC is a highly heterogeneous disease, and the role of MELK across different TNBC subtypes warrants further investigation. Comprehensive genomic and proteomic analyses of primary patient samples may identify additional pathways or co-regulated genes that contribute to MELK-driven metastasis. Moreover, the potential interaction of MELK with other therapeutic targets, such as FOXM1 and the NF-κB pathway, should be explored to identify combinatorial treatment strategies.

In conclusion, our findings underscore the potential of MELK as a therapeutic target in TNBC. The reduction in tumorigenic and metastatic properties following MELK inhibition supports the rationale for developing MELK-targeted therapies. While pharmacologic inhibitors like OTSSP167 have shown efficacy in preclinical models, their off-target effects must be carefully addressed in future studies. The development of MELK-specific inhibitors with improved selectivity and reduced toxicity could provide a more effective therapeutic strategy for patients with TNBC. This, coupled with more comprehensive mechanistic studies, will be essential to translate these findings into clinical benefits for patients with TNBC.

## 4. Materials and Methods

### 4.1. Cell Culture

The basal-like BC cell lines MDA-MB-231 and BT-549 were grown from frozen samples (American Type Culture Collection [ATCC]). MDA-MB-231 cells were grown in DMEM (Gibco, Fisher Scientific, Waltham, MA USA) supplemented with 10% FBS (Gibco, Fisher Scientific) and 1% penicillin/streptomycin (Gibco, Fisher Scientific). BT-549 cells were grown in RPMI 1640 (Gibco, Fisher Scientific) with 10% FBS (Gibco, Fisher Scientific) and 1% penicillin/streptomycin (Gibco, Fisher Scientific). All cell lines were grown in a 5% CO_2_ incubator. Cells were passaged at approximately 70% confluence. Cells were tested for mycoplasma routinely (MycoAlert, Lonza, Basel, Switzerland).

### 4.2. Stable Cell Line Generation, Transfections, siRNAs, and Plasmids

For transfection, two Eppendorf tubes were labeled for each sample and one control set: one tube for Lipofectamine^®^ reagent mixture and the other for DNA and P3000™. A total of 125 µL of Opti-MEM Reduced Serum Medium was added to each tube. Each transfection required 5 µL of Lipofectamine^®^ 3000 reagent, added to the reagent tube. For DNA, 2.5 µg was added to the DNA tube along with 5 µL of P3000™ reagent. The DNA and P3000™ mixture was transferred to the corresponding reagent tube and was incubated at room temperature for at least 5 min. During incubation, the current media was removed from each well in the 6-well plate, and 1.75 mL of antibiotic-free media was added to each well. After incubation, the transfection mixture was mixed again and 250 µL was added to each designated well in droplets, followed by gentle swirling to mix. The plate was returned to the incubator. Cells were replaced with complete media after 24 h.

For knockdown experiments, cells were seeded in 6-well plates and transfected with 100 nM ON-TARGET plus SMARTpool siRNA (ThemoScientific) targeting MELK or non-targeting control (Non-targeting Pool, catalogue no. D-001810-10-50, using oligofectamine (Invitrogen, Waltham, MA USA) according to the manufacturer’s instructions). The following are the catalogue numbers and the siRNA sequences: ON-TARGET plus Human MELK SMARTpool, catalogue no. J-004029-06, J-004029-09, set# LQ-004029-00-0002, target sequences, #1 GA and #4 GG. Cells were trypsinized and seeded 24 h post-transfection and used in clonogenic survival assays as well as for RNA extractions to determine the knockdown efficiency.

shRNA construct and stable clone generation: The pTRIPZ lentiviral system with MELK inducible shRNA transfection starter kit was purchased from ThermoScientific using catalog #RHS4696-200703132 and cat# RHS4696-200691582 for non-template control and shMELK. Stable cell lines were generated using lentiviral transduction. Lentiviral particles with shMELK or non-targeting shControl were packaged and culture medium provided at the vector core facility of University Michigan. Stable cell lines were selected using complete medium containing puromycin at 2 ug/mL for one-week selection. Clones were selected and screened for both RFP and MELK expression changes and were used as pools and as selected stable clones in all in vitro and in vivo experiments.

### 4.3. Flow Cytometry

Samples for flow cytometry were prepared using standard protocols. Cells (1 × 10^6^) were trypsinized, filtered, and resuspended in PBS with 3% FBS. For staining, cells were allocated into FACS tubes labeled “Stain”, “DEAB”, and “Isotype”. Compensation controls were prepared in tubes labeled “Unstained”, “ALDH”, “PE-Cy7”, “APC”, and “DAPI”. Staining master mixes were prepared using FITC-Aldefluor (1 μL/sample), PE-Cy7-CD24 (1 μL/sample), APC-CD44 (20 μL/sample), and DAPI (0.2 μL/sample). Corresponding isotype controls included PE-Cy7-Isotype (4 μL/sample) and APC-Isotype (2 μL/sample).

Cells were incubated with staining or isotype mixes at 37 °C for 30–60 min, resuspending every 15 min. Following incubation, cells were washed, centrifuged at 4 °C for 5 min at 300–350 g, and resuspended in Aldefluor staining buffer or PBS with 3% FBS. Samples were analyzed immediately and kept on ice until analysis. Compensation tubes were prepared similarly, with reagents specific to the respective fluorophores.

### 4.4. Western Blot Analysis

For protein isolation, cells from tissue culture lines were washed once with ice-cold phosphate-buffered saline (PBS) and lysed in a protein lysis buffer containing 50 mM HEPES (pH 7.5), 150 mM NaCl, 1 mM EDTA, 1% Triton X-100, 10% glycerol, 100 mM NaF, Complete Mini protease inhibitor cocktail (Roche, Basel, Switzerland), and phosphatase inhibitor cocktails I and II (Sigma-Aldrich, St. Louis, MO, USA). Protein concentration was measured using the BCA Protein Assay (Pierce Biotechnology, Rockford, IL, USA). For western blot analysis, 30 μg of total protein was resolved on a 10% SDS-PAGE gel and transferred to a nitrocellulose membrane (Amersham Biosciences, Buckinghamshire, UK). The membrane was blocked, incubated with primary antibodies, washed with TBST, then incubated with horseradish peroxidase-conjugated secondary antibodies. Antigen–antibody complexes were detected using the ECL or ECL Plus chemiluminescent system (Amersham Biosciences). Primary antibodies for MELK (Sigma Prestige, Catalog #HPA017214), cleaved PARP (Cell Signaling, Catalog #5625S, Danvers, MA, USA), and total PARP (Cell Signaling, Catalog #9542S) were used. Anti-rabbit secondary antibodies were obtained from Sigma. For protein isolation from human tumor samples, the same protocol was followed with an additional homogenization step. Tumor samples were homogenized using a 7 mm generator and a rotator–stator homogenizer (ProScientific, Oxford, CT, USA) in protein lysis buffer, and all isolation procedures were performed on ice.

### 4.5. qPCR

Total RNA was isolated using TRIzol (Invitrogen) and RNeasy kit (Qiagen, Hilden, Germany) according to manufacturers’ instruction. Total RNA was reverse transcribed into cDNA using SuperScript III and primers (Invitrogen). Quantitative PCR (qPCR) was performed using SYBR Green Master Mix (Applied Biosystems, Waltham, MA USA) on an Applied Biosystems 7900HT Real-Time System. The relative quantity of the target gene was computed for each sample using the ΔΔCt method by comparing the mean Ct of the gene to the mean Ct of the housekeeping gene GAPDH. All the primers were obtained from Integrated DNA Technologies (IDT). Results were reported as average expression ± standard error of the mean. Primer sequences were as follows: MELK forward #1: 5′ ->3′ GCT GCA AGG TAT AAT TGA TGGA MELK reverse #1: 5′ -> 3′ CAG TAA CAT AAT GAC AGA TGG GC.

### 4.6. Mammosphere Formation Assay

For pharmacologic inhibition experiments, MDA-MB-231 cells were treated with 100 nM OTSSP 167. For the shRNA knockdown experiments, MDA-MB-231 cells were transfected with either control non-targeting shRNA or MELK-targeting shRNA. For each experiment, non-stem (CD44+/CD24−/ALDH−) and stem-like (CD44−/CD24−/ALDH+) cells were subsetted by FACS using anti-CD-24-PE and anti-CD44-fluorescein isothiocyanate (FITC) monoclonal antibodies. The number of viable cells per ml was calculated using trypan blue. Cells were resuspended in 0.5 mL of complete mammosphere media in each well of a precoated 24-well ultra-low adherent plate with a seeding density of 120–150 cells/per well. For the shRNA knockdown experiments, there were both doxycycline-positive and doxycycline-negative plates. Cells were seeded on ultra-low-attachment 96-well plates at 37 °C and 5% CO_2_ for 5–10 days (depending on the cell line and the size of mammospheres) without disturbing the plates, particularly during the growth phase (the first 5 days). After the culture period, the mammospheres were counted under a light microscope at 10× magnification. Mammosphere-forming efficiency (MFE%) was calculated using the following equation: MFE% = (# of mammospheres per well)/(# of cells seeded per well) × 100.

### 4.7. Modified Invasion Assay

Diluted Matrigel (1–2 mg/mL in serum-free media) was added to 24-well transwell inserts (100 μL/well) and gelled at 37 °C for 5 h. BT-549 or MDA-MB-231 cells were harvested 48 h post-siRNA transfection, washed in 1% FBS media, and resuspended at 1–2 × 10^6^ cells/mL. Cell suspensions (100 μL) were seeded onto the Matrigel layer.

The lower chamber was filled with 600 μL of media containing 10% FBS, and transwells were incubated at 37 °C for 24–48 h. Inserts were then washed, fixed in 70% ethanol, stained with 0.09% crystal violet, and washed. Stained cells were dissolved in 10% acetic acid, and absorbance was measured at 560 nm to assess invasion. Images were taken at 40× magnification.

### 4.8. Invasion Assay

Matrigel (1–2 mg/mL in serum-free media) was added to the upper chambers of 24-well transwell inserts (100 μL/well) and gelled at 37 °C for 5 h. Inserts were placed into companion plates containing chemoattractant media. T47D control and T47D MELK-GFP-overexpressing cells (4 × 10^4^ cells in 100 μL complete culture media) were seeded into the upper chambers. The lower chambers were filled with complete culture media as a chemoattractant. Cells were fixed with 70% ethanol before doubling time. Invading cells were quantified using a fluorescence microscope at 4× magnification after 24 h, and invasion efficiency was calculated as follows: % Invasion = (Number of cells in lower chamber/Total number of cells seeded in the upper chamber) × 100.

### 4.9. Scratch-Wound Assay

Cells (50,000/well) were seeded in ImageLock 96-well plates (Sartorius, Singapore) and incubated overnight at 5% CO_2_. Wounds were created using the WoundMaker Tool (Sartorius). For MDA-MB-231 cells, wells were washed twice with dPBS to remove detached cells, while washing was omitted for BT-549 cells to preserve wound clarity. Media containing 1 μL/mL DMSO (control), OTSSP167 at 100 nM, or OTSSP167 at 1 μM was added. Plates were scanned every 3 h in an Incucyte system (Sartorius) until control wells reached confluence (48 h for MDA-MB-231; 72 h for BT-549).

### 4.10. Chorioallantoic Membrane (CAM) Assay

The CAM assay was performed as previously described [60]. Fertilized chicken eggs were incubated at 37.5 °C and 65% humidity for 6 days. Cell suspensions (25 μL containing 1.5 × 10^6^ T47D LacZ control or T47D MELK-overexpressing cells) were grafted near the allantoid vein bifurcation without touching the CAM. Eggs were incubated for 18 days, after which tumors were excised, weighed, and the embryonic liver and lungs were harvested for human cell detection via quantitative human Alu-specific PCR. DNA quantification was performed using fluorogenic TaqMan qPCR probes and DNA copy number were quantified as described [61].

### 4.11. Drug Information

OTSSP167 (HY-15512A) was purchased from MedChemExpress (Princeton, NJ, USA).

### 4.12. Statistical Analyses

Statistical analyses were performed in R (version 4.2.2), except the scratch-wound assay, which was conducted in Prism (https://www.graphpad.com/features, accessed on 20 February 2025). A significance level threshold of *p* < 0.05 was used. Statistical tests and sample sizes for each assay are specified in the figure captions. Statistical analyses were derived from a sample size of at least three unless otherwise indicated.

### 4.13. Kaplan–Meier Analysis

Survival analyses for overall survival (OS), recurrence-free survival (RFS), and distant metastasis-free survival (DMFS) were performed using the Kaplan–Meier Plotter database. This dataset is comprised of 7830 unique tumor samples from 55 independent datasets. Clinical characteristics for this dataset, which have been described, include receptor status, grade, lymph node status, molecular subtype distribution, applied treatment, and length of follow-up for RFS [18]. Breast cancer patient cohorts were stratified into high- and low-*MELK* expression groups based on median *MELK* expression levels. The gene symbol “MELK” was used to query the database, and the survival metrics OS, RFS, and DMFS were selected for analysis. Results were visualized as Kaplan–Meier survival curves.

### 4.14. Transcriptomics Analysis

RNA-seq data for samples from primary tumors were collected from the TCGA-BRCA database, https://portal.gdc.cancer.gov/projects/TCGA-BRCA (accessed on 20 June 2023) using TCGAbiolinks (version 2.26.0) [62]. The TCGA workflow for transcriptomic analysis was used for gene expression analysis [63]. Data were normalized using dataNorm and low-expression genes were filtered using dataFilt using the “quantile” with a cutoff of 0.25. To identify genes whose expression was correlated with *MELK*, the Hmisc function rcorr was used, with default parameters. A cutoff of *p* < 0.05 was used to determine significantly correlated genes. This was followed by overrepresentation analysis on the top 100 positively correlated and top 100 negatively correlated genes with the R package fgsea (version 1.24.0), using an adjusted *p*-value cutoff of *p* < 0.05 [64].

## Data Availability

The data presented in this study are available upon request from the corresponding author.

## Supplementary Materials

The supporting information can be downloaded at https://www.mdpi.com/article/10.3390/ijms26052245/s1.

## Abbreviations

The following abbreviations are used in this manuscript:

TNBC	Triple-negative breast cancer
MELK	Maternal embryonic leucine zipper kinase
BCSC	Breast cancer stem cell
OS	Overall survival
RFS	Recurrence-free survival
DMFS	Distant metastasis-free survival
KEGG	Kyoto Encyclopedia of Genes and Genomes
ORA	Overrepresentation Analysis
MFE	Mammosphere Formation Efficiency
OE	Overexpression
CAM	Chorioallantoic Membrane
