# Supplementary material for: MELK as a Mediator of Stemness and Metastasis in Aggressive Subtypes of Breast Cancer
Source: Int J Mol Sci. 2025 Mar 3;26(5):2245. doi: 10.3390/ijms26052245 (PMC11900306; doi:10.3390/ijms26052245)
Supplement: Supplementary file 1 [file ijms-26-02245-s001.zip › ijms-3480055-supplementary.pdf]

Supplemental Table 1. Genes with expression positively correlated to MELK expression in TCGA-BRCA samples

Gene Symbol	Pearson's r	p	Gene Symbol	Pearson's r	p
CEP55	0.806	<1e-16	***MTFR2***	0.709	<1e-16
KIFC1	0.794	<1e-16	***BUB1B***	0.709	<1e-16
TPX2	0.793	<1e-16	***KIF14***	0.707	<1e-16
KIF4A	0.788	<1e-16	***NEIL3***	0.705	<1e-16
NCAPG	0.785	<1e-16	***STMN1***	0.705	<1e-16
HJURP	0.783	<1e-16	***DEPDC1B***	0.703	<1e-16
TTK	0.783	<1e-16	***MSH2***	0.703	<1e-16
CENPA	0.774	<1e-16	***SGO2***	0.701	<1e-16
CKAP2L	0.774	<1e-16	***ORC1***	0.700	<1e-16
DLGAP5	0.774	<1e-16	***DNA2***	0.699	<1e-16
SKA1	0.774	<1e-16	***CHEK1***	0.699	<1e-16
BUB1	0.773	<1e-16	***SUV39H2***	0.699	<1e-16
NDC80	0.772	<1e-16	***CKS1B***	0.698	<1e-16
NCAPH	0.771	<1e-16	***NUF2***	0.698	<1e-16
CDCA8	0.766	<1e-16	***SPC25***	0.697	<1e-16
KIF20A	0.762	<1e-16	***MASTL***	0.696	<1e-16
KIF2C	0.758	<1e-16	***NCAPG2***	0.696	<1e-16
KIF11	0.756	<1e-16	***GAS2L3***	0.696	<1e-16
ERCC6L	0.755	<1e-16	***CDC7***	0.695	<1e-16
DBF4	0.754	<1e-16	***PLK4***	0.695	<1e-16
KIF15	0.751	<1e-16	***SPDL1***	0.694	<1e-16
CCNB2	0.748	<1e-16	***EZH2***	0.693	<1e-16
ARHGEF39	0.744	<1e-16	***CKS2***	0.692	<1e-16
CENPI	0.743	<1e-16	***CENPF***	0.691	<1e-16
KIF23	0.740	<1e-16	***GINS1***	0.689	<1e-16
CENPO	0.740	<1e-16	***CDCA7***	0.687	<1e-16
RAD54L	0.739	<1e-16	***MCM3***	0.687	<1e-16
STIL	0.738	<1e-16	***CDC20***	0.687	<1e-16
IQGAP3	0.736	<1e-16	***MCM6***	0.687	<1e-16
SGO1	0.734	<1e-16	***CDC45***	0.686	<1e-16
POLQ	0.732	<1e-16	***VRK1***	0.685	<1e-16
CLSPN	0.731	<1e-16	***FAM83D***	0.685	<1e-16
CCNA2	0.729	<1e-16	***FOXM1***	0.684	<1e-16
PLK1	0.725	<1e-16	***TRIP13***	0.683	<1e-16
SKA3	0.724	<1e-16	***FAM72B***	0.681	<1e-16
KIF18B	0.724	<1e-16	***CDCA2***	0.680	<1e-16
KIF18A	0.724	<1e-16	***AUNIP***	0.680	<1e-16
DONSON	0.722	<1e-16	***RAD51***	0.679	<1e-16
HASPIN	0.719	<1e-16	***OIP5***	0.679	<1e-16
AURKB	0.719	<1e-16	***RACGAP1***	0.678	<1e-16
EXO1	0.718	<1e-16	***RFC4***	0.678	<1e-16
CENPL	0.718	<1e-16	***NEK2***	0.677	<1e-16
GTSE1	0.717	<1e-16	***PUM3***	0.675	<1e-16
CDC25A	0.716	<1e-16	***CIP2A***	0.675	<1e-16
LMNB1	0.715	<1e-16	***ARHGAP11A***	0.675	<1e-16
MKI67	0.715	<1e-16	***UBE2C***	0.674	<1e-16
CENPE	0.713	<1e-16	***TROAP***	0.674	<1e-16
CENPN	0.712	<1e-16	***FAM72A***	0.674	<1e-16
ASPM	0.712	<1e-16	***GPSM2***	0.673	<1e-16
CCNB1	0.710	<1e-16	***PDSS1***	0.672	<1e-16

Supplemental Table 2. Genes with expression negatively correlated to MELK expression in TCGA-BRCA samples

Gene Symbol	Pearson's r	p-value	Gene Symbol	Pearson's r	p-value
TNS2	-0.330	<1e-16	***GPIHBP1***	-0.245	<1e-16
CIRBP	-0.327	<1e-16	***ENSG00000270179***[1]	-0.245	<1e-16
NOSTRIN	-0.308	<1e-16	***ENSG00000237529***[1]	-0.244	<1e-16
REEP6	-0.308	<1e-16	***BCAM***	-0.244	<1e-16
RAPGEF3	-0.305	<1e-16	***TMEM204***	-0.244	<1e-16
MLPH	-0.289	<1e-16	***IZUMO4***	-0.244	2.220e-16
SLC27A1	-0.285	<1e-16	***CYB561D2***	-0.243	2.220e-16
CFAP99	-0.283	<1e-16	***NEIL1***	-0.243	2.220e-16
CCDC159	-0.282	<1e-16	***ENSG00000245468***[1]	-0.243	2.220e-16
LTC4S	-0.279	<1e-16	***TMEM25***	-0.242	4.441e-16
DEGS2	-0.278	<1e-16	***NHLRC4***	-0.241	4.441e-16
PHYHD1	-0.277	<1e-16	***NPDC1***	-0.241	4.441e-16
RAB17	-0.273	<1e-16	***HPGDS***	-0.241	4.441e-16
DYNLRB2	-0.267	<1e-16	***ENSG00000280339***[1]	-0.240	4.441e-16
ACBD4	-0.267	<1e-16	***UBXN10***	-0.240	4.441e-16
CFAP69	-0.266	<1e-16	***DNAAF1***	-0.240	4.441e-16
FAM47E	-0.264	<1e-16	***ARHGEF38***	-0.239	4.441e-16
DRC3	-0.263	<1e-16	***EFCAB12***	-0.239	4.441e-16
SHC2	-0.261	<1e-16	***CPLX1***	-0.239	4.441e-16
SCN2B	-0.261	<1e-16	***CLDN5***	-0.239	6.661e-16
MEIS3	-0.260	<1e-16	***TNFSF12***	-0.239	6.661e-16
PODN	-0.260	<1e-16	***CST3***	-0.239	8.882e-16
CBX7	-0.260	<1e-16	***PLD4***	-0.238	8.882e-16
LYRM9	-0.259	<1e-16	***EFCAB6***	-0.237	1.110e-15
NOP53	-0.258	<1e-16	***AVPR2***	-0.237	1.332e-15
ANKRD24	-0.256	<1e-16	***KLHDC1***	-0.235	1.998e-15
BBS5	-0.255	<1e-16	***SPEF1***	-0.235	1.998e-15
ENSG00000254040[1]	-0.254	<1e-16	***COL14A1***	-0.235	2.220e-15
TGFB3	-0.253	<1e-16	***LDLRAD2***	-0.235	2.442e-15
CYB5D2	-0.253	<1e-16	***APH1B***	-0.234	3.109e-15
HSPA12B	-0.253	<1e-16	***SUSD3***	-0.233	3.109e-15
RAI2	-0.252	<1e-16	***NXNL2***	-0.233	3.109e-15
MFAP4	-0.251	<1e-16	***HPN***	-0.233	3.553e-15
CRY2	-0.251	<1e-16	***MIR3936HG***	-0.233	3.997e-15
LAMP5	-0.251	<1e-16	***ABLIM3***	-0.233	3.997e-15
LAMB2	-0.251	<1e-16	***SSC5D***	-0.232	4.441e-15
DNAAF8	-0.251	<1e-16	***CCDC96***	-0.232	4.663e-15
RHOB	-0.250	<1e-16	***TMEM91***	-0.232	5.329e-15
ZNF843	-0.249	<1e-16	***TTC36***	-0.232	5.329e-15
C3orf18	-0.249	<1e-16	***ZNF688***	-0.232	5.329e-15
PCSK4	-0.248	<1e-16	***PDGFD***	-0.232	5.329e-15
GAMT	-0.248	<1e-16	***SEMA3G***	-0.232	5.329e-15
TMC4	-0.247	<1e-16	***PALM***	-0.231	5.551e-15
LINC00993	-0.247	<1e-16	***NUDT18***	-0.231	5.773e-15
KIF13B	-0.247	<1e-16	***MXD4***	-0.231	7.105e-15
PGPEP1	-0.246	<1e-16	***ENSG00000259793***[1]	-0.230	7.772e-15
NTN4	-0.246	<1e-16	***LRRN4CL***	-0.230	7.994e-15
NPAS1	-0.245	<1e-16	***C4A***	-0.230	9.326e-15

ENSG00000230882	-0.245	<1e-16	*PLAC9*	-0.230	9.548e-15
CYBRD1	-0.245	<1e-16	*SPARCL1*	-0.229	1.021e-14

ENSEMBL ID used instead of genes that have no symbols.[1]

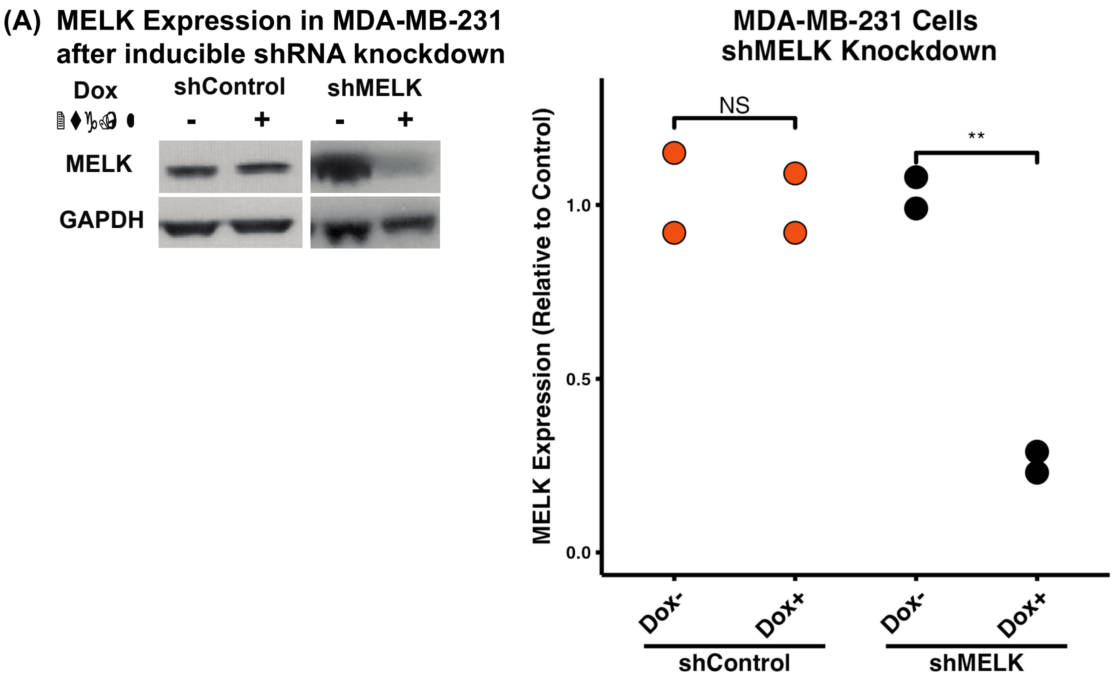

Supplemental Figure 1. Successful MELK knockdown with inducible shRNA in MDA-MB-231 cells. (**A**) Western blot of doxycycline (Dox)-inducible shRNAs against MELK shows shMELK reduces MELK protein expression. On the other hand, ShControl does not reduce MELK protein expression, making it an effective control shRNA. (**B**) Quantification of the expression of MELK with control and MELK shRNAs via qPCR shows significant reduction in expression only after induction of shMELK (n = 2, p = 0.008), and not in shControl (n = 2, p = 0.857). Data represent two independent experiments. A 2-sided Student's t-test was used for comparison. **p < 0.01, NS p >= 0.05.

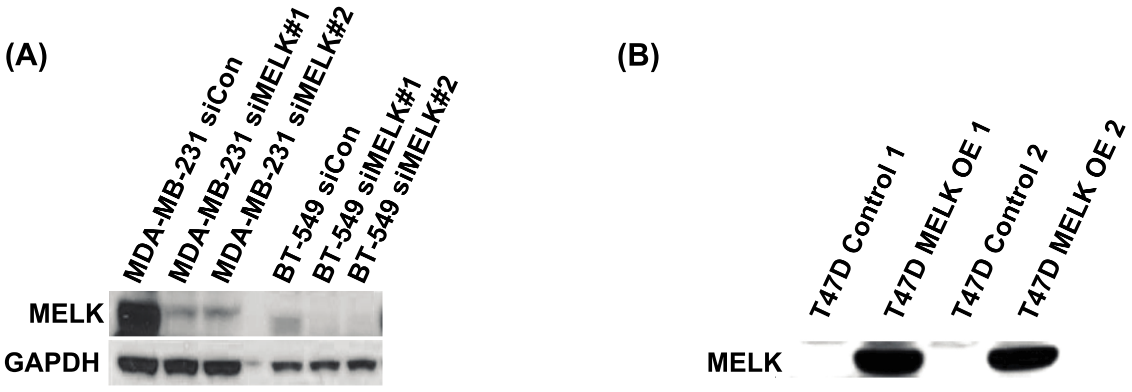

Supplemental Figure 2. Successful knockdown (with siRNA) and overexpression of MELK. (**A**) Western blot showing reduced MELK expression in two colonies with an siRNA against MELK, siMELK#1 and siMELK#2, in the high-MELK-expressing cell lines MDA-MB-231 and BT-549, compared to MELK expression with a control siRNA, siCon. (**B**) Western blot showing increased MELK expression in two colonies (MELK OE 1 and MELK OE 2) of the low-MELK expressing cell line, T47D, compared to controls (Control 1 and Control 2). OE: Over-expressing.

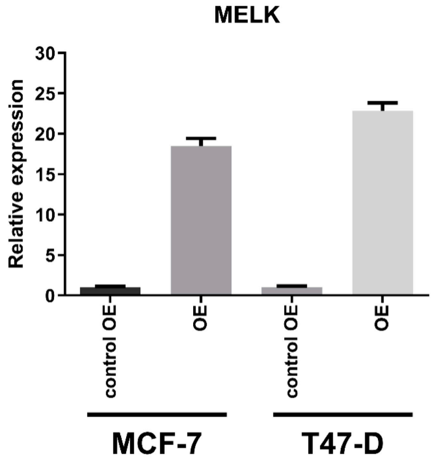

Supplemental Figure 3. *MELK* over-expression in low-MELK-expressing cell lines MCF7 and T47D. qRT-PCR analysis shows successful over-expression of *MELK* in MCF7 and T47D compared to controls. Data represent the mean of three independent experiments, with error bars indicating SEM. OE: Over-expressing.
